# Age-Related Differences in Reorganization of Functional Connectivity for a Dual Task with Increasing Postural Destabilization

**DOI:** 10.3389/fnagi.2017.00096

**Published:** 2017-04-12

**Authors:** Cheng-Ya Huang, Linda L. Lin, Ing-Shiou Hwang

**Affiliations:** ^1^School and Graduate Institute of Physical Therapy, College of Medicine, National Taiwan UniversityTaipei, Taiwan; ^2^Physical Therapy Center, National Taiwan University HospitalTaipei, Taiwan; ^3^Institute of Physical Education, Health and Leisure Studies, National Cheng Kung UniversityTainan, Taiwan; ^4^Institute of Allied Health Sciences, College of Medicine, National Cheng Kung UniversityTainan, Taiwan; ^5^Department of Physical Therapy, College of Medicine, National Cheng Kung UniversityTainan, Taiwan

**Keywords:** aging, EEG, dual task, functional connectivity, balance control

## Abstract

The aged brain may not make good use of central resources, so dual task performance may be degraded. From the brain connectome perspective, this study investigated dual task deficits of older adults that lead to task failure of a suprapostural motor task with increasing postural destabilization. Twelve younger (mean age: 25.3 years) and 12 older (mean age: 65.8 years) adults executed a designated force-matching task from a level-surface or a stabilometer board. Force-matching error, stance sway, and event-related potential (ERP) in the preparatory period were measured. The force-matching accuracy and the size of postural sway of the older adults tended to be more vulnerable to stance configuration than that of the young adults, although both groups consistently showed greater attentional investment on the postural task as sway regularity increased in the stabilometer condition. In terms of the synchronization likelihood (SL) of the ERP, both younger and older adults had net increases in the strengths of the functional connectivity in the whole brain and in the fronto-sensorimotor network in the stabilometer condition. Also, the SL in the fronto-sensorimotor network of the older adults was greater than that of the young adults for both stance conditions. However, unlike the young adults, the older adults did not exhibit concurrent deactivation of the functional connectivity of the left temporal-parietal-occipital network for postural-suprapostural task with increasing postural load. In addition, the older adults potentiated functional connectivity of the right prefrontal area to cope with concurrent force-matching with increasing postural load. In conclusion, despite a universal negative effect on brain volume conduction, our preliminary results showed that the older adults were still capable of increasing allocation of neural sources, particularly via compensatory recruitment of the right prefrontal loop, for concurrent force-matching under the challenging postural condition. Nevertheless, dual-task performance of the older adults tended to be more vulnerable to postural load than that of the younger adults, in relation to inferior neural economy or a slow adaptation process to stance destabilization for scant dissociation of control hubs in the temporal-parietal-occipital cortex.

## Introduction

Maintenance of postural balance requires attentional resources corresponding to the degree of postural threat (Remaud et al., 2012). Postural response requires complexity and numerous micro-adjustments, and stable bilateral stance is principally regulated by an automatic process using brainstem synergy (Honeycutt et al., 2009). Increasing postural destabilization shifts postural control to a more controlled process involving the frontal and cortical-basal ganglia loop (Jacobs and Horak, 2007; Boisgontier et al., 2013). Due to the additional attentional investment, postural response becomes more regular in the controlled process (Donker et al., 2007; Stins et al., 2009). Central resource allocation of a postural-suprapostural dual-task is an elaborate trade-off, flexibly depending on response compatibility of the two subtasks. Addition of a secondary task (or suprapostural task; Mitra and Fraizer, 2004) to a postural task does not necessarily result in dual-task degradation due to resource competition (Chen and Stoffregen, 2012; Stoffregen, 2016); instead, postural response can be integrated with suprapostural activity to facilitate suprapostural performance (Stoffregen et al., 1999; Prado et al., 2007). On the other hand, although a great number of the studies conducted on dual tasks have employed two cognitive tasks, very few neuroimaging studies have focused on postural-suprapostural dual tasks because of methodological constraints. With the event-related potential (ERP) of scalp electroencephalogram (EEG), Huang and Hwang (2013) reported that the amplitudes of the N1 and P2 waves in the preparation period prior to executing a secondary motor task varied with task loads of the postural and suprapostural tasks, respectively. The N1 amplitude reflected anticipatory arousal and postural response preceding the force-matching (Adkin et al., 2008; Mochizuki et al., 2008; Sibley et al., 2010; Huang et al., 2014). An increasing N1 amplitude in the sensorimotor and parietal areas implies more attentive control required for postural destabilization (Huang and Hwang, 2013; Little and Woollacott, 2015). On the other hand, P2 amplitude related to neural resource for visuomotor processing of the subsequent force-matching event, with a greater P2 amplitude associated with a less task load of force-matching (Huang and Hwang, 2013; Hwang and Huang, 2016).

In older adults, the shrinkage of a wide range of cortical areas causes evolving dysfunction of a dual task (Fernandes et al., 2006; Hartley et al., 2011). Degeneration of the frontal-parietal network specifically impairs executive processes keyed to dual tasking, such as response inhibition, task switching (Cole et al., 2013), and selective attention to relevant information (Mozolic et al., 2011). Age-related dual task deficits also manifest with a resource ceiling (Geerligs et al., 2014) and compensatory recruitment of additional brain resources (Hartley et al., 2011; Boisgontier et al., 2013), especially when the dual task places high demands on those resources. Behavioral studies have shown that postural destabilization can multiply the dual task cost for the elderly of a postural-suprapostural task. Past researches employing a choice reaction time task (Shumway-Cook and Woollacott, 2000) and digit 2-back task (Rapp et al., 2006; Doumas et al., 2008) highlighted age-related differences in suprapostural performance by increasing stance instability rather than degrading visual stimulation of the suprapostural task. In effect, the brain's residual capacity wanes with aging, which causes negative postural penetrability to suprapostural processing. Hence, older adults often adopt a postural prioritization strategy to keep attentional resources on the postural task (Lacour et al., 2008; Liston et al., 2014). However, behavioral data that elucidate the neural correlates of resource allocation in older adults during a postural-suprapostural task are very limited. Although, neural evidence of age-related deficits has been extensively sought using classic dual tasks, the findings up to date cannot be directly applied to postural-suprapostural dual tasks on account of issues of response compatibility (Salo et al., 2015). For instance, the task quality of a suprapostural motor task such as juggling or tray-carrying takes advantage of stance stability (Balasubramaniam et al., 2000; Wulf et al., 2004; McNevin et al., 2013), whereas parallel loading of two cognitive tasks always causes mutual interference. To our knowledge, no studies have investigated alterations in information transfer for a postural-suprapostural task performed by older adults, despite the degeneration of the white matter integrity of the brain with aging (Furst and Fellgiebel, 2011; de Groot et al., 2016). Hence, it is worthwhile to characterize the differences in the functional connectivity of the frontal/prefrontal areas to other cortical regions [such as the parietal (Gontier et al., 2007) and premotor areas (Marois et al., 2006)] of young and older adults during a postural-suprapostural task.

A challenging postural set-up is a sensitive way to highlight age-related differences in a postural-suprapostural task. To explore the underlying neural mechanisms of dual-task interference of a postural-suprapostural task, we investigated age effect on ERP dynamics for force-matching from the level-surface to stabilometer stances during the preparatory period of the particular dual task. Defined as the time window between the execution beep and onset of the force-matching act, the preparatory period consists of posture-dependent N1 and supraposture-dependent P2 waves that encrypt cognitive processing of pre-movement stance regulation, task switching from the posture subtask to supraposture subtask, and planning of the subsequent force-matching act (Huang and Hwang, 2013; Huang et al., 2014; Hwang and Huang, 2016). For the young healthy adults, a postural-suprapostural task with increasing postural instability caused reorganization of functional connectivity in the preparatory period with anterior shift of processing resources and dissociation of control hubs in the parietal-occipital cortex (Huang et al., 2016). Within the brain connectome context, this study aimed to extend on previous work by exploring differences in the component amplitudes (N1 and P2) and functional connectivity of the ERP in young and older adults during the performance of a suprapostural motor task with increasing postural challenge. This study hypothesized that, with increasing postural load, young adults would exhibit smaller changes than older adults in the component amplitudes of ERP (N1 and P2) and functional connectivity, especially those for the fronto-parietal network in the preparatory period. We also hypothesized that topological reorganization of functional connectivity due to increasing postural load would differ in the two populations.

## Materials and methods

### Subjects

Twelve young healthy adults (5 female and 7 male, age: 25.25 ± 1.25 years, range 21–33 years) and 12 older healthy adults (5 female and 7 male, age: 65.83 ± 1.01 years, range: 61–73 years) participated in this study. Subjects were volunteers from the local community and university campus who responded to a poster or a network advertisement. All of the participants were right-handed and had no history of neurological or musculoskeletal diagnoses. The older adults in this study, who had regular exercise habits, had experienced no falls in the previous 6 months. They participated in the postural-suprapostural experiment after signing personal consent forms approved by the local ethics committee (University Hospital, National Cheng Kung University, Taiwan).

### Procedures

Before the main experiment, each participant was instructed to stand on a stabilometer in a shoulder-width stance with their arms hanging by their sides. The stabilometer was a wooden platform (50 × 69 cm) with a curved base (height: 18.5 cm). When the platform of the stabilometer was in the horizontal position, the midline of the platform (34.5 cm from the front/rear edge) passed through the anterior aspect of the participant's bilateral lateral malleolus. The positions of the participant's feet were used in the following experiment. Then the maximal angle of anterior tilt was determined from the readings of an electrogoniometer (Model SG110, Biometrics Ltd., UK; output accuracy: 1 mv = 0.09 degrees) on the ankle joint as the participants tilted the stabilometer with maximum plantarflexion of the ankle joint. In addition, we determined the force of each participant's maximum voluntary contraction (MVC) from three attempts of the right thumb-index precision grip during quiet upright stance. The stabilometer is commonly used to train balance in clinics and provides postural challenge for single postural task (Wulf et al., 2001; McNevin et al., 2003; Chiviacowsky et al., 2010) and postural-suprapostural dual-task in the laboratories (Wulf et al., 2003; Huang et al., 2014, 2016; Hwang and Huang, 2016). Therefore, we used the stabilometer to produce postural destabilization in this study.

**Figure 1:**
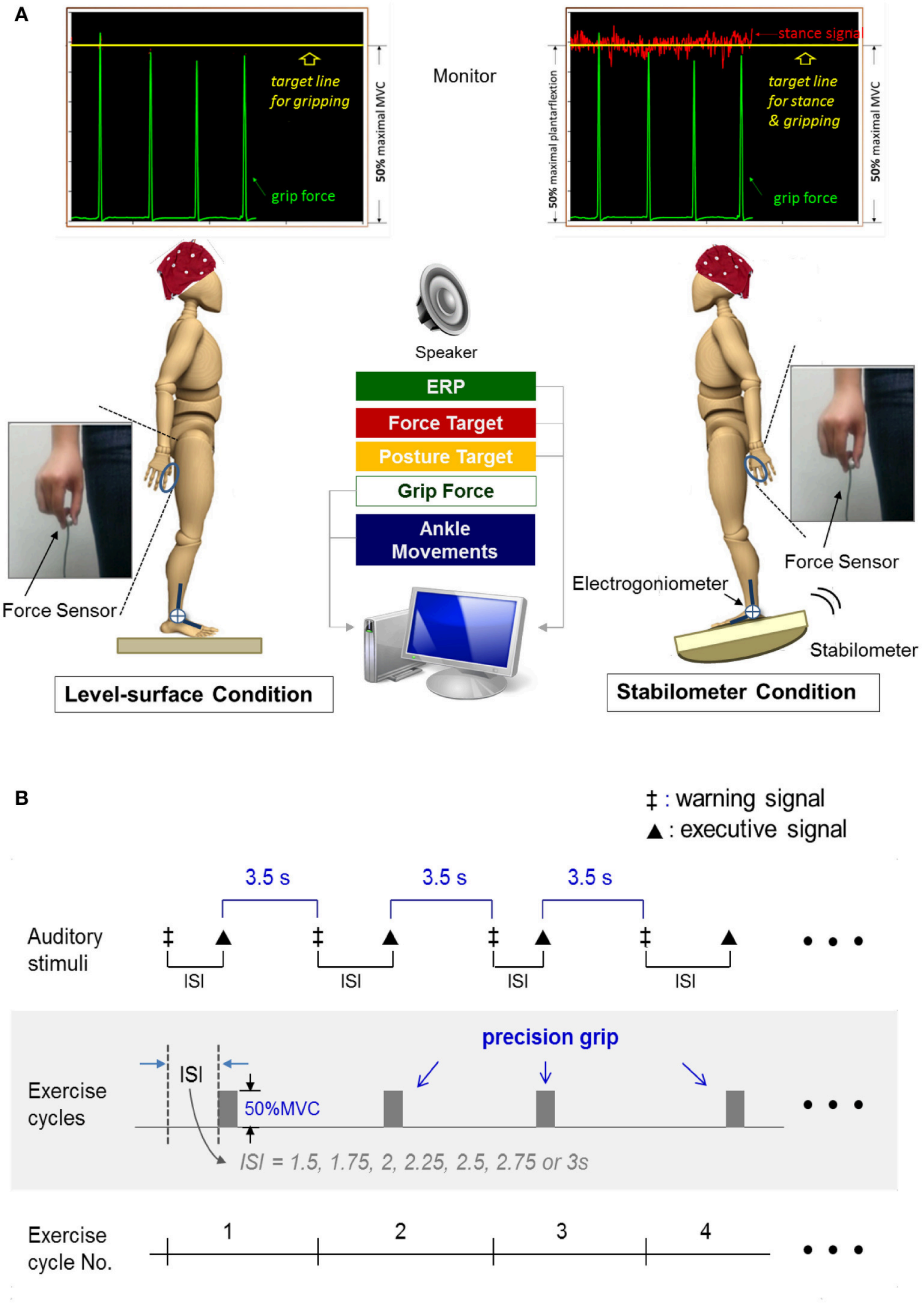
**Schematic illustration of experimental setup (A)** and auditory stimulus paradigm for force-matching task **(B)**.

The formal experiment required the participants to conduct a dual task (suprapostural force-matching and postural tasks) with on-line visual feedback under two different randomized stance conditions (level-surface vs. stabilometer). A monitor that displayed force output, ankle movement, and the target signals was placed 60 cm in front of the subject at eye-level. The subject conducted a thumb-index precision grip to couple a target line of 50% MVC force (pre-determined in the experiment) and concurrently maintained a stable upright stance with minimal ankle movement on a wooden level surface or a tilted stabilometer. Participants were not told to prioritize either task, and they were instructed to perform both postural and force-matching tasks as well as possible. The stabilometer produced less postural disturbance than was used in our previous studies (Hwang and Huang, 2016) because the balance capacity of the elderly participants was poorer than that of the young adults. The postural task in the level-surface and stabilometer conditions required the participants to couple the ankle joint angle derived from the readings of the electrogoniometer to the target line, based on visual feedback. The target lines for the postural task in the level-surface and stabilometer conditions were set at the horizontal surface and 50% of the maximal anterior tilt, respectively (Figure 1A). The postural tasks are known as postural tasks of visual internal focus (Huang et al., 2014), with which the participants should control upright stance with ankle angular displacement (or an internal aspect of body movement). Utilization of an internal focus for a postural task will interfere with postural automatic processes, especially when difficulty is added to stance control in a dual task condition for the elderly (Chiviacowsky et al., 2010). To minimize the potential visual load during the concurrent tasking, the target signals for posture and force-matching were carefully scaled at the same vertical position of the monitor for each participant (Figure 1A). We fully understood that the relative task difficulty of the postural and suprapostural tasks was a critical determinant of the reciprocal effect of the postural-suprapostural task. An earlier pilot experiment had shown that the present dual task setup would not significantly degrade the force-matching accuracy of the young adults between the level-surface and stabilometer conditions (Hung et al., 2016). In this particular dual task design, stance destabilization was expected to produce a decline in force-matching performance due to increasing postural threat (stabilometer vs. level-surface) in the older participants (Boisgontier et al., 2013). With this design, we were able to examine the age effect on the compensatory mechanisms underlying perseverance of quality of the secondary motor task when balance contexts varied.

Execution of the suprapostural force-matching in an experimental trial was first cued by a warning signal (an 800 Hz tone lasting for 100 ms). Upon hearing an executive tone (a 500 Hz tone lasting for 100 ms), the participants then started a quick thumb-index precision grip (force impulse duration <0.5 s) to couple instantaneously the peak precision-grip force with the force target on the monitor. The warning-executive signal pairs were randomly presented at different intervals of 1.5, 1.75, 2, 2.25, 2.5, 2.75, or 3 s (Figure 1B). The interval between the end of the executive tone and the beginning of the next warning tone was 3.5 s. There were a total of 14 warning-executive signal pairs in an experimental trial (80 s per trial) and six experimental trials of the postural-motor dual task for each stance condition. Both young and older subjects were allowed for a fixed rest duration between trials (1 min) to minimize fatigue effect.

### Experimental setting

A 40-channel NuAmps amplifier (NeuroScan Inc., EI Paso, TX, USA) with Ag-AgCl scalp electrodes was used to record scalp voltage fluctuations from different 30 EEG channels (Fp_1/2_, F_z_, F_3/4_, F_7/8_, FT_7/8_, FC_z_, FC_3/4_, C_z_, C_3/4_, CP_z_, CP_3/4_, P_z_, P_3/4_, T_3/4_, T_5/6_, TP_7/8_, O_z_, and O_1/2_). The ground electrode was placed along the midline ahead of F_*z*_. Electrodes placed above the arch of the left eyebrow and below the eye were used to monitor eye movements and blinks. The impedances of all the electrodes were below 5 kΩ and were referenced to linked mastoids of both sides. The EEG data was recorded with a band-pass filter set at 0.1–100 Hz and with a sampling rate of 1 kHz. The electrogoniometer was attached to the dominant ankle joint to record the angular motion of the ankle joint. The electrogoniometer consisted of two sensors. One sensor was placed at the dorsum of the right foot between the second and third metatarsal heads, and the other sensor was fastened along the midline of the middle third of the anterior aspect of lower leg. A load cell (15-mm diameter × 10-mm thickness, net weight = 7 g; Model: LCS, Nippon Tokushu Sokki Co., Japan) on the right thumb was used to record the level of force-matching. All physiological data were synchronized and digitized at a sampling rate of 1 kHz in LabVIEW software (National Instruments, Austin, TX, USA).

### Data analyses

#### Behavior data

**Figure 2:**
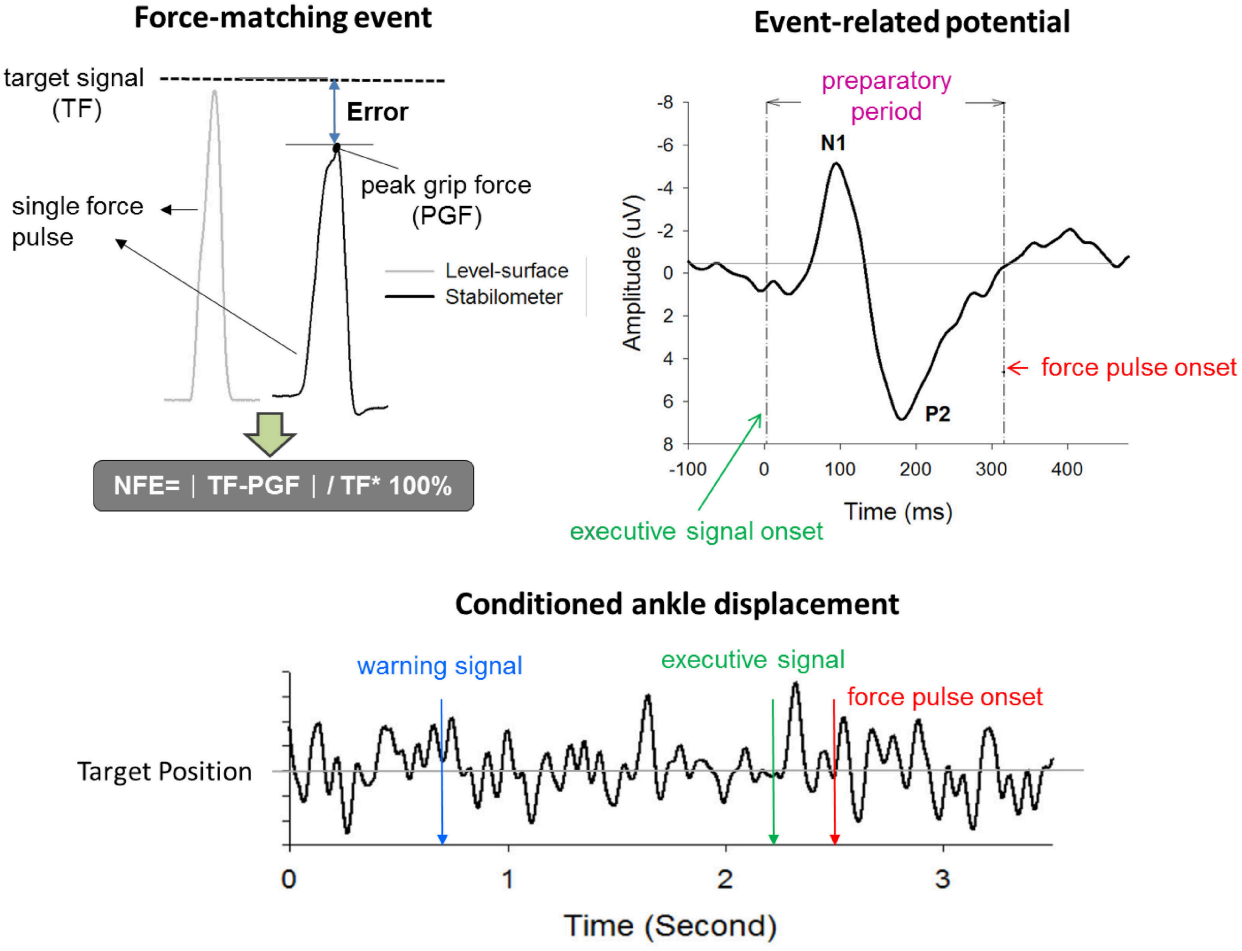
**Real-time display of precision grip force, ankle displacement, and target signals for concurrent force-matching and postural tasks**. To reduce the visual load during the experiment, the target signals of both postural and force-matching tasks were displayed in an identical position on the monitor, by separate scale-tuning of the manual force target and postural target. Force-matching performance was assessed with normalized force error (NFE). The event-related potential (ERP) associated with force-matching was registered with scalp electroencephalography. ERP between the executive tone and the onset of the force-impulse profile was denoted as the preparatory period. ERP in this period primarily contained N1 and P2 components. The ankle displacement after conditioning with a low-pass filter is labeled by three critical events (warning signal, executive tone, and force-impulse onset). TF, target force; PGF, peak grip force.

Normalized force error (NFE) of force-matching was used to represent suprapostural performance in the present study. Force-matching error was represented in terms of NFE, or $\frac{\left|\text{TF}-\text{PGF}\right|}{\text{TF}}\times 100\%$ (PGF: peak grip force; TF: target force; Figure 2). The NFEs of all force-matching events were averaged across trials for each subject in the level-surface and stabilometer conditions. The reaction time (RT) of force-matching was denoted as the timing interval between the executive tone and the onset of grip force. Postural performance was characterized with the fluctuation properties of ankle movement during the interval between the warning signal and the onset of force pulse. We applied root mean square (RMS) and sample entropy (SampEn) to assess the amplitude and complexity of the ankle movement fluctuations (AMF_RMS and AMF_SampEn) after resampling the kinematic data to 125 Hz. SampEn is an appropriate entropy measure for reliably quantifying the variability structure of biological data with a short length (Yentes et al., 2013). The mathematical formula for SampEn was

$$\begin{array}{l} SampEn\left(m,r,N\right)=-\text{log}(\frac{\sum_{i=1}^{N-m}A_{i}}{\sum_{i=1}^{N-m}B_{i}}) \end{array}$$

where *r* = 15% of the standard deviation of the ankle movement fluctuations, *m* is the length of the template (*m* = 3), and *N* is the number of data points in the time series. *Ai* is the number of matches of the *i*th template of length *m* + 1 data points, and *Bi* is the number of matches of the *i*th template of length m data points. A SampEn close to 0 represents greater periodicity (or regularity), while a value near 2 represents higher complexity (or irregularity). Higher regularity (or lower SampEn value) of postural sway represents the more attentional focus being paid to postural control, and vice versa (Donker et al., 2007; Borg and Laxåback, 2010; Kuczyński et al., 2011).

#### Component amplitudes and functional connectivity of multi-channel ERP

ERP data was analyzed off-line with the NeuroScan 4.3 software program (NeuroScan Inc., EI Paso, TX, USA). Prior to ERP quantitative analysis, third-order trend correction and eye movement correction protocols were applied to the entire set of recorded data to remove the DC shift and eye movement artifacts. The eye movement artifacts were removed from the EEG using regression analysis (Semlitsch et al., 1986), and the number of eye blinks in each trial was roughly 10–15 across subjects. After eye movement was removed, the EEG data were conditioned with a low-pass filter (40 Hz/48 dB roll-off), and then the conditioned EEG data were segmented into epochs of 700 ms, including 100 ms before the onset of each execution signal. Epochs were all baseline-corrected at the pre-stimulus interval. Poor epochs, such as those affected by excessive drift or eye blinks, were discarded by visual inspection (rejection rate of inappropriate trials: <8%). The remaining artifact-free epochs were averaged for an experimental trial in the level-surface and stabilometer conditions, and then the ERP data were also grouped according to a two-factor design (population: the young and older adults; postural task: level-surface and stabilometer stances).

As postural-suprapostural behaviors involve information mastery dependent upon the fronto-motor-parietal network (Huang and Hwang, 2013), we expected age-related differences in regional activity of ERP due to increasing difficulty of the postural subtask of the dual task in the global frontal (GF: Fp_1_, Fp_2_, F_3_, F_z_, F_4_, F_7_, and F_8_), sensorimotor (SM: C_3_, C_z_, C_4_, CP_3_, CP_z_, and CP_4_), and parietal-occipital (PO: P_3_, P_z_, P_4_, O_1_, O_z_, and O_2_) areas for the level-surface and stabilometer conditions. The N1 and P2 amplitudes were quantified as the peak amplitude in two separate time windows (80–150 ms, 150–240 ms after executive signal onset). The ERP of each electrode contained N1 and P2 components, which were selectively averaged to obtain amplitudes of the N1 and N2 of the above-mentioned areas. For instance, amplitudes of N1 and P2 recorded from the electrodes of the Fp_1_, Fp_2_, F_3_, F_z_, F_4_, F_7_, and F_8_ were averaged to represent the size of N1 and P2 of the global frontal area.

Based on multi-channel ERP signal, we also quantified statistical interdependencies of non-stationary ERP in the preparatory period with one of the most popular approaches, synchronization likelihood (SL). The SL measures the degrees of linear and non-linear dimensions of EEG/MEG coupling within cortical networks (Leistedt et al., 2009; Boersma et al., 2011). Theoretically, SL takes into account the recurrences of state space vectors occurring at the same moment that are converted from two time-series of interest (Stam et al., 2005). SL can sensitively detect slight variations in the coupling strength for a fine time scale (Stam and van Dijk, 2002), which is appropriate for resolve ERP synchronization patterns in a short period. An SL close to 0 indicates no coupling; an SL of 1 indicates complete coupling. For brevity, detailed descriptions of SL calculation (Stam and van Dijk, 2002; Stam et al., 2003) and parameter settings (Montez et al., 2006) can be found in previous works. Computation of the SL across all pairs of ERP data of the channels in the preparatory phase (the time interval between the executive tone and the force-matching onset) produced a square 30 × 30 SL adjacent matrix. Each entry in the SL adjacent matrix represented the connectivity strength within the functional networks. For each participant, the overall SL adjacent matrix from all experimental trials in the level-surface or stabilometer condition was averaged. SL thresholds from 0.1 to 0.9 were selected to build functional connectomes of different connection strengths. The SL adjacent matrix was rescaled with the proportion of strongest weights, such that all other weights below a given threshold (including SL on the main diagonal) were set to 0. Namely, the selection of the SL threshold of 0.1 merely accounted for the strongest 10% of the weights in the SL adjacent matrix (or functional connectivity in the functional connectome). SL was calculated with the functions of HERMES for Matlab (Niso et al., 2013). The mean value of SL for all the electrode pairs was defined as SL_All. The mean values of SL that connected to the specified areas, the fronto-sensorimotor (SL_FSM), and parietal-occipital (SL_PO) areas, were determined for the level-surface and stabilometer conditions.

#### Statistical analyses

The purpose of this study was to examine the neural mechanisms underlying age and stance effects on postural-suprapostural performance. The current experimental design focused on the neural mechanisms responsible for differential stance effects on force-matching accuracy between young and older adults. Two way repeated measures ANOVA with population (young and older) and postural load (level-surface and stabilometer) were used to examine the significance of differences in behavior parameters (NFE, RT, AMF_RMS, and AMF_SampEn), and the mean SL of the areas of interest (SL_All, SL_FSM, and SL_PO) across different threshold values. The level of significance of the above-mentioned statistical analyses was set at *p* = 0.05. The significance of the *post-hoc* test for stance and age effects was *p* = 0.0125 using the Bonferroni correction. Moreover, network-based statistics (NBS) were performed to vigorously identify stance-related changes in the functional connectivity of all the node pairs for the young and older groups. For each group, paired *t*-tests were independently performed at each synchronization value, and *t*-statistics larger than an uncorrected threshold of *t*_(13)_ = 3.012 (*p* = 0.005) were extracted into a set of supra-threshold connections. Then we identified all connected components in the adjacency matrix of supra-threshold links and saved the number of links. A permutation test was performed 5,000 times to estimate the null distribution of the maximal component size, and the corrected *p*-value was calculated as the proportion of permutations for which the most connected components consisted of two or more links. Methodological details of NBS are documented in Zalesky et al. (2010). The age effect on the topological distribution of significant stance-related differences in synchronization value were examined with visual inspection. Statistical analyses were performed in Matlab (Mathworks Inc. Natick, MA, USA) and SPSS v.19.0 (SPSS Inc. Chicago, IL, USA). All data are presented as mean ± standard error.

## Results

### Behavior performance

**Figure 3:**
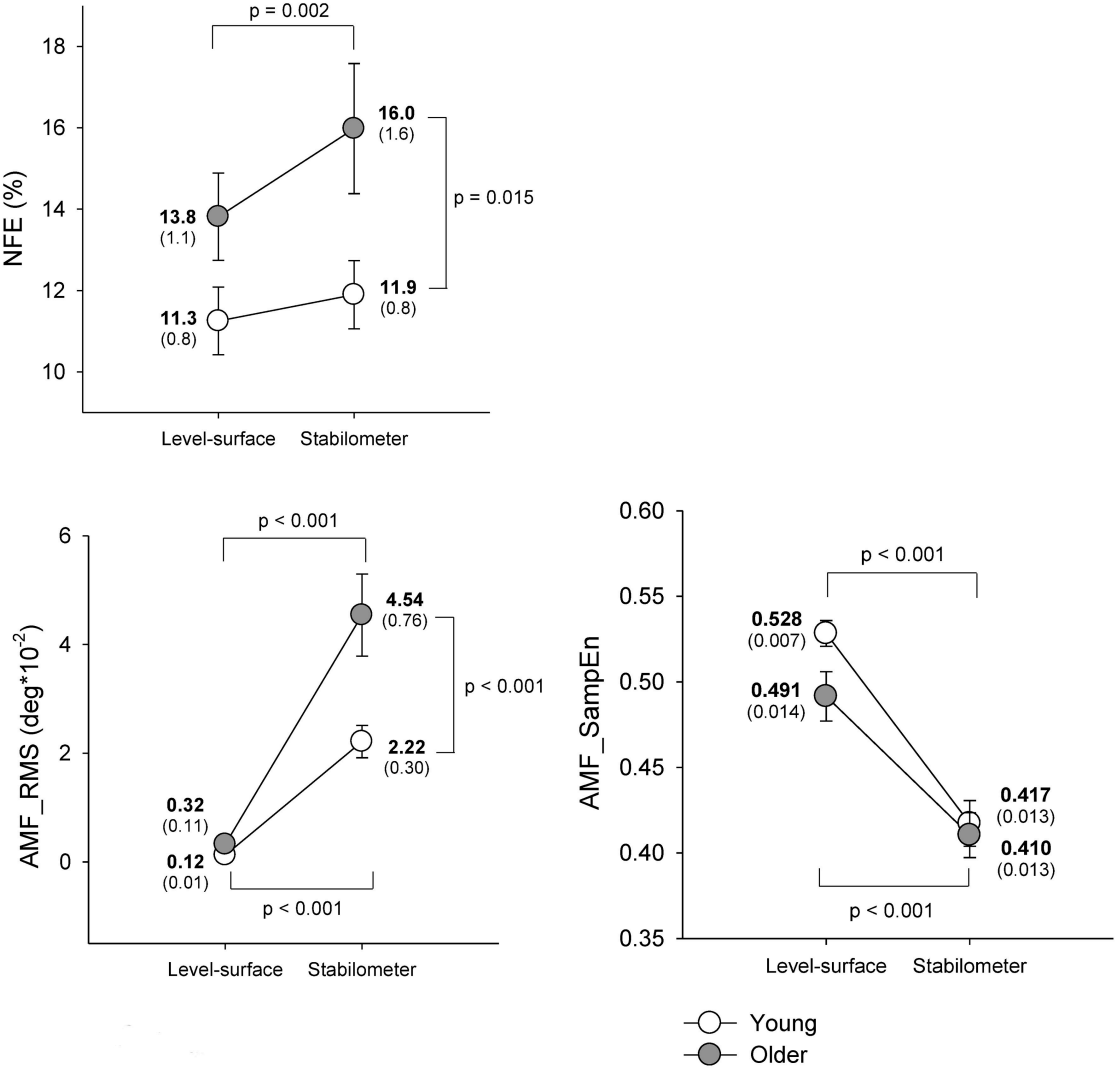
**Means and standard errors of force-matching and postural variables for the concurrent force-matching and postural tasks under the level-surface and stabilometer conditions for young and older populations**. NFE, normalized force error; AMF_RMS, root mean square value of ankle fluctuation movements; AMF_SampEn, sample entropy of ankle fluctuation movements.

Figure 3 shows means and standard errors of task performance of force-matching and postural response for young and older groups under the level-surface and stabilometer conditions. The ANOVA results revealed that NFE was subject to both stance and age effects [stance: *F*_(1, 22)_ = 10.36, *p* = 0.004; age: *F*_(1, 22)_ = 4.60, *p* = 0.043; stance × age: *F*_(1, 22)_ = 3.04, *p* = 0.095]. On account of a marginal interaction effect, we continued the *post-hoc* analysis which indicated that NFE of the older group was more susceptible to stance configuration and the older adults performed worse force-matching in the stabilometer condition than in the level-surface condition (*p* = 0.002). In contrast, NFE of the young group was not affected by stance configuration (*p* = 0.307). The RT of the force-matching was not age dependent [*F*_(1, 22)_ = 1.57, *p* = 0.223], but varied with stance pattern [*F*_(1, 22)_ = 4.55, *p* = 0.044] without a significant interaction [*F*_(1, 22)_ = 1.06, *p* = 0.315; Young: level-surface = 307.2 ± 7.8 ms, stabilometer: 326.4 ± 5.6 ms; Older: level-surface = 303.0 ± 8.3 ms, stabilometer: 308.9 ± 4.5 ms]. In terms of RMS, the magnitude of ankle movement fluctuations was also a function of age and stance configuration [stance: *F*_(1, 22)_ = 67.22, *p* < 0.001; age: *F*_(1, 22)_ = 8.50, *p* = 0.008; stance × age: *F*_(1, 22)_ = 7.63, *p* = 0.011]. *Post-hoc* analysis revealed that both the young and the older adults exhibited greater ankle movement fluctuations during the stabilometer stance than during surface stance (*p* < 0.001). In particular, the ankle movement fluctuations of the older adults were greater than those of the young adults in the stabilometer condition (*p* < 0.001). In addition, irregularity of the ankle movement fluctuations was subject to stance configuration rather than to age effect [stance: *F*_(1, 22)_ = 73.46, *p* < 0.001; age: *F*_(1, 22)_ = 2.62, *p* = 0.120; stance × age: *F*_(1, 22)_ = 1.79, *p* = 0.125]. Increases in stance difficulty resulted in a consistently lower AMF_SampEn (more regularity) of the ankle movement fluctuations in the young and older groups (*p* < 0.001).

### ERP component amplitude

**Figure 4:**
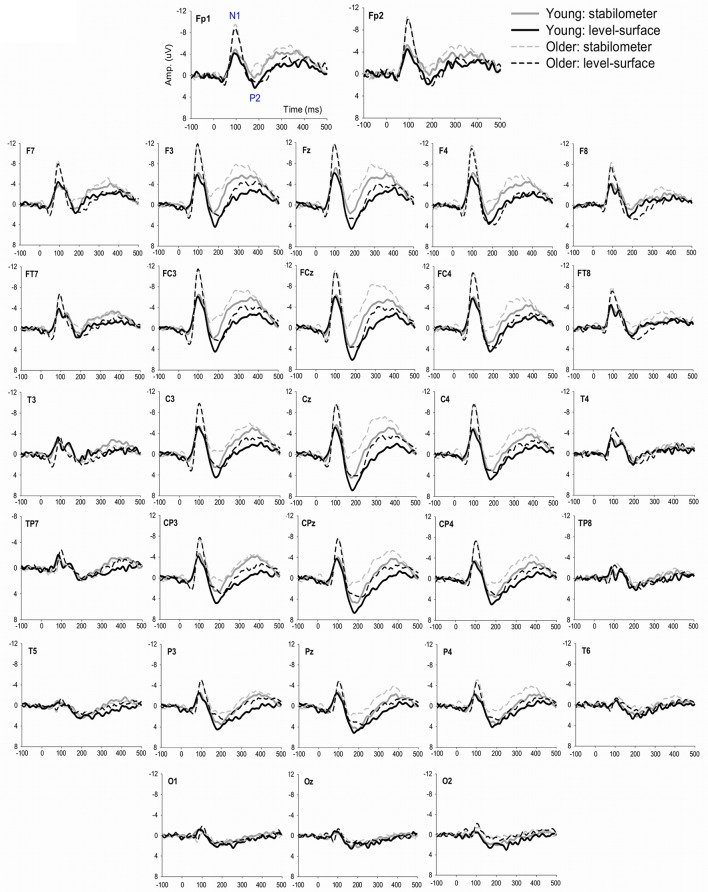
**The contrast of pooled ERP profile between the level-surface and stabilometer conditions for the young and older populations**.

**Figure 5:**
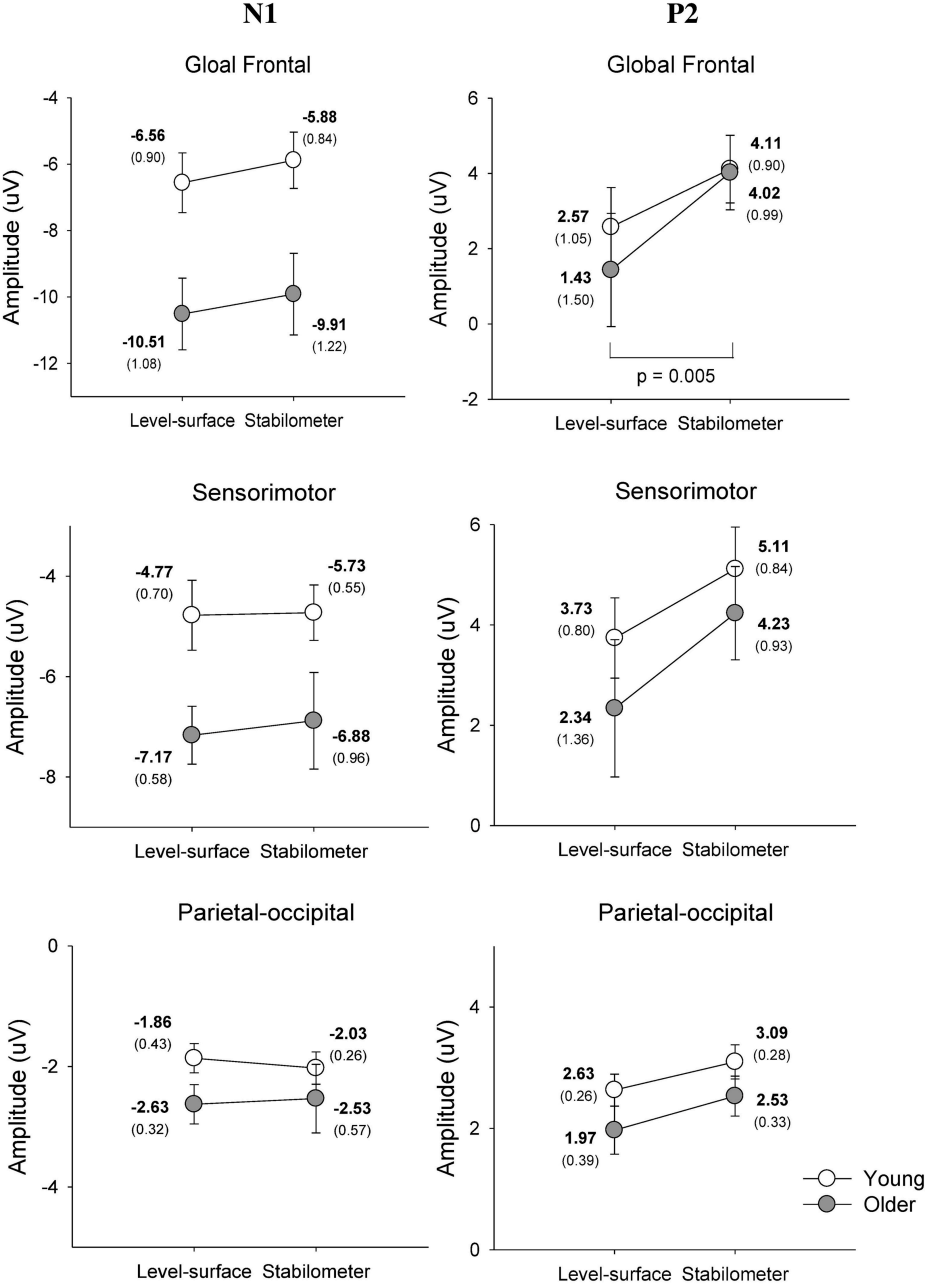
**Mean amplitudes of the N1 and P2 components of the ERP in the global frontal, sensorimotor, parietal-occipital areas**.

Figure 4 show pooled ERP profiles of the each electrode of the young and older adults in the level-surface and stabilometer conditions. Stance-related differences in the ERP profiles were evident in the anterior portions of the cortex, irrespective of the populations. The N1 amplitude in the GF and SM areas varied significantly with age [GF: *F*_(1, 22)_ = 8.14, *p* = 0.009; SM: *F*_(1, 22)_ = 5.54, *p* = 0.028], but not with stance [GF: *F*_(1, 22)_ = 2.74, *p* = 0.112; SM: *F*_(1, 22)_ = 0.31, *p* = 0.582] or interaction effects [GF: *F*_(1, 22)_ = 0.01, *p* = 0.919; SM: *F*_(1, 22)_ = 0.15, *p* = 0.699]. However, N1 amplitude of the PO areas did not significantly vary with stance and age effects [stance: *F*_(1, 22)_ = 0.03, *p* = 0.860; age: *F*_(1, 22)_ = 1.66, *p* = 0.211; stance × age: *F*_(1, 22)_ = 0.44, *p* = 0.513]. In contrast, the P2 amplitudes in the GF, SM, and PO areas were all dependent on stance configuration [GF: *F*_(1, 22)_ = 12.32, *p* = 0.002; SM: *F*_(1, 22)_ = 13.37, *p* = 0.001; PO: *F*_(1, 22)_ = 6.03, *p* = 0.022], rather than on age [GF: *F*_(1, 22)_ = 0.17, *p* = 0.683; SM: *F*_(1, 22)_ = 0.71, *p* = 0.408; PO: *F*_(1, 22)_ = 2.30, *p* = 0.143] or interaction effects [GF: *F*_(1, 22)_ = 0.79, *p* = 0.382; SM: *F*_(1, 22)_ = 0.34, *p* = 0.566; PO: *F*_(1, 22)_ = 0.05, *p* = 0.818; Figure 5].

### Functional connectivity of ERP in the preparatory phase

**Figure 6:**
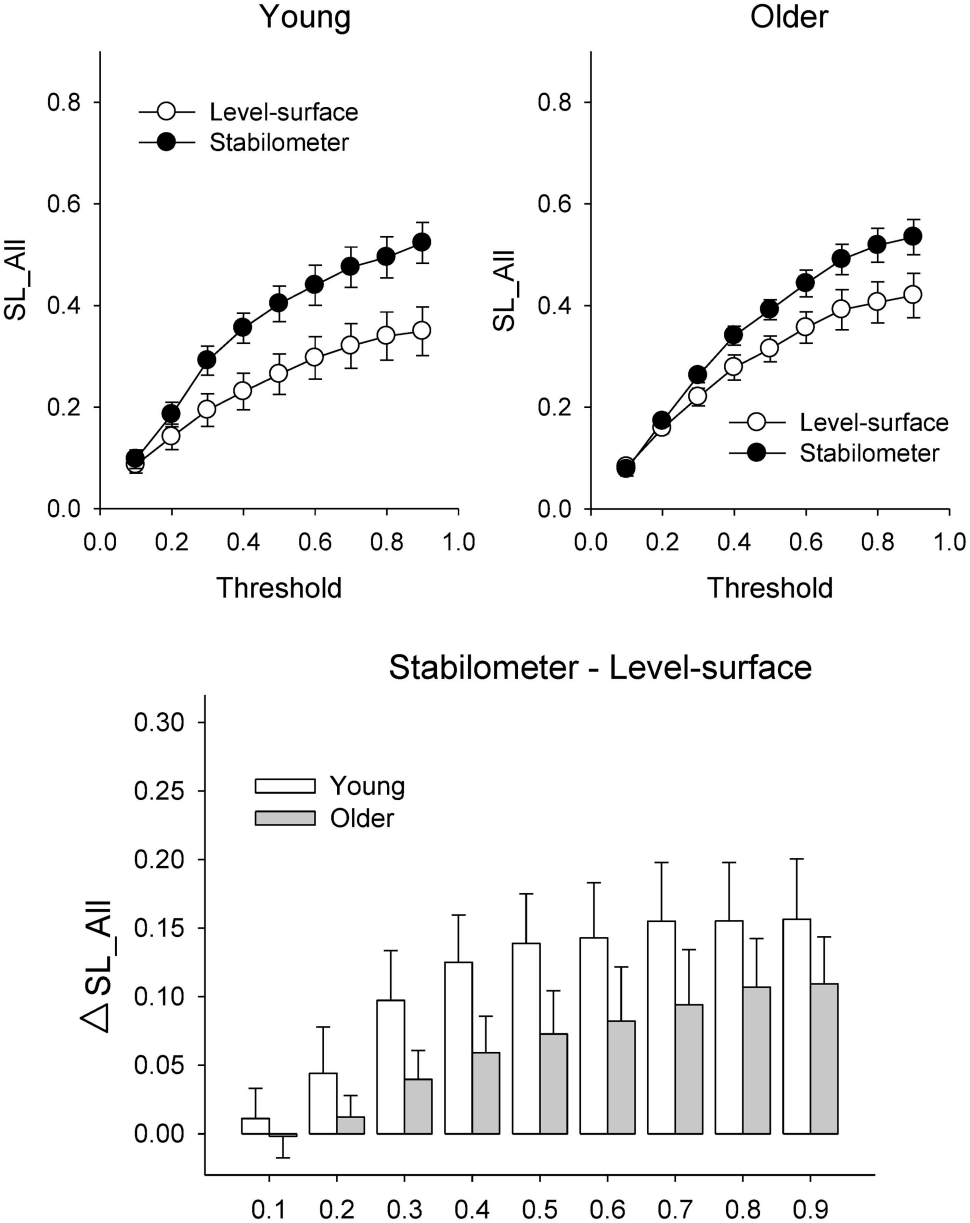
**Population means of SL of all electrode pairs (SL_All) across all threshold values in the surface-level and stabilometer conditions (refer to Table **1**)**. The stance-related differences in SL (stabilometer—level-surface) across various threshold values are highlighted for the young and older groups in the lower plot. At the threshold values of 0.3–0.9, there was a significant stance effect on SL_All, with increasing SL_All for the stabilometer condition for young and older adults.

**Figure 7:**
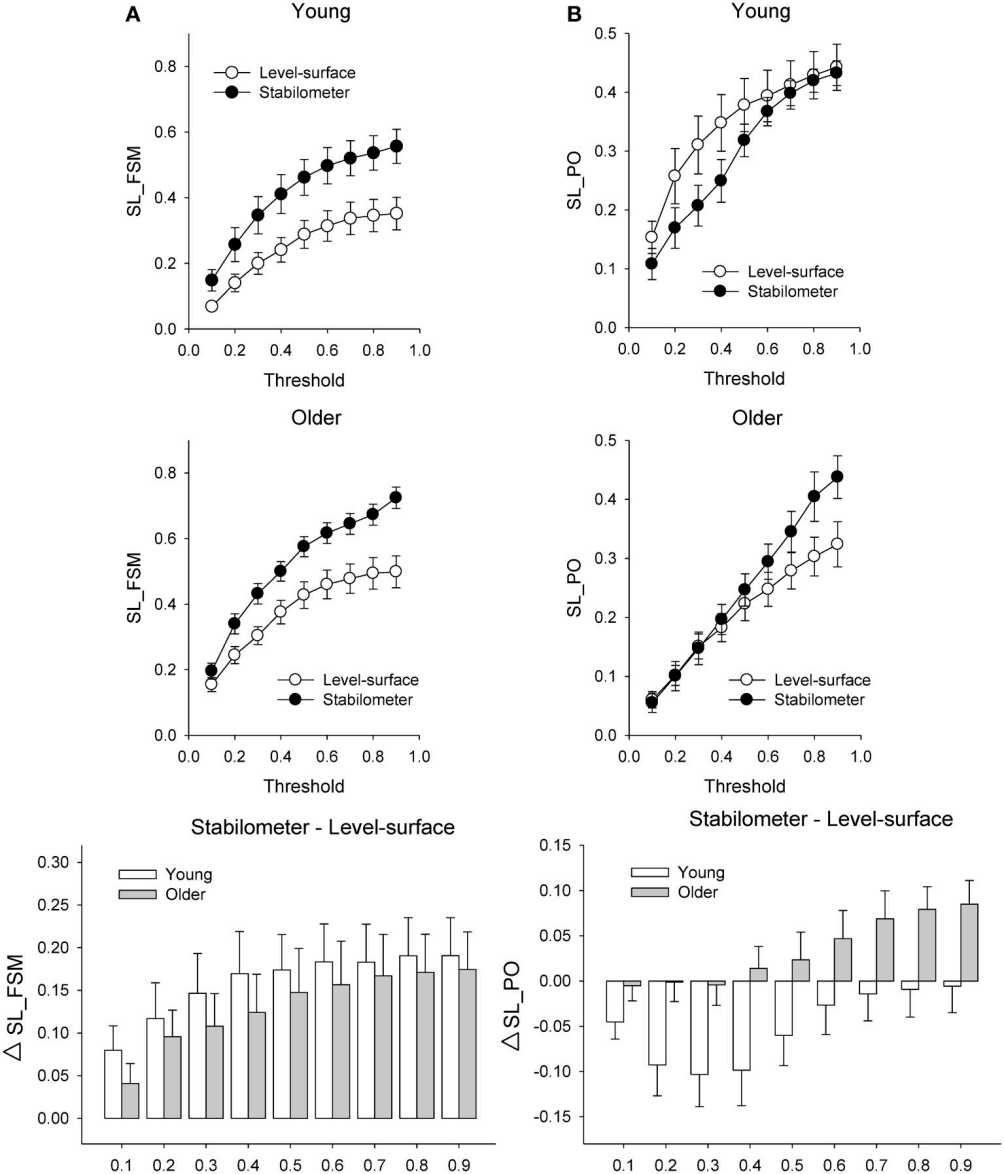
**Population means of SL of all electrode pairs in the fronto-sensorimotor (FSM, refer to Table **2**) (A)** and parietal-occipital (PO, refer to Table **3**) **(B)** networks across all threshold values in the surface-level and stabilometer conditions. The stance-related differences in SL (stabilometer—level-surface) across various threshold values are highlighted for the young and older groups in the lowest plots. With increasing stance difficulty from level-surface to stabilometer, there were significant age and stance effects on SL_FSM for all threshold values, except that age effect on SL_FSM was marginally significant at the threshold value of 0.2.

Figure 6 presents the mean SL (SL_All) of all electrode pairs in the level-surface and stabilometer conditions and stance-related change in SL (ΔSL_All) as a function of threshold value. For the both groups, SL_All tended to be larger in the stabilometer condition than in the level-surface condition. Table 1 shows the detailed results of ANOVA for age and stance effects on SL_All across different thresholds. For thresholds of 0.1 and 0.2, main effects of stance and age on SL_All were not significant (*p* > 0.05). For thresholds of 0.3–0.9, SL_All was subject to a main effect of stance, and SL was significantly larger in stabilometer condition than in the level-surface condition (*p* < 0.05). Figure 7A presents the mean SL of the electrode pairs in the fronto-sensorimotor network (SL_FSM) for the level-surface and stabilometer conditions and stance-related change in SL (ΔSL_FSM) as a function of threshold value. Table 2 summarizes the ANOVA results for age and stance effects on SL_FSM across different thresholds. For all threshold values, SL_FSM varied with age and stance configuration (*p* < 0.05), except for a marginal effect of age for a threshold setting of 0.2. That was, the SL_FSM of the young and older adults increased in the stabilometer condition for all threshold values (*p* < 0.006), and the older adults exhibited a larger SL_FSM than the young adults in the level-surface and stabilometer conditions (*p* < 0.05). The most remarkable difference in SL modulation for stance difficulty increment between the young and older groups was in the PO area (Figure 7B). Table 3 summarizes the ANOVA results for age and stance effects on SL_PO across different thresholds. For threshold values of 0.2–0.4, SL_PO was significantly subject to the interaction effect of age and stance configuration (*p* < 0.05). For the young adults, *post-hoc* analysis further showed that SL_PO in the stabilometer condition was smaller than that in the level-surface condition (*p* < 0.0125). Notably, such a stance-dependent decline in SL_PO at lower threshold value was not present in the older group (*p* > 0.0125). Interaction effect of age and stance configuration on SL_PO for the threshold values of 0.8 and 0.9 was also significant (*p* < 0.05). Particularly at the threshold value of 0.9, *post-hoc* analysis revealed that the SL_PO for the older adults potentiated with increasing postural load (*p* = 0.009), but not the SL_PO of the young adults (*p* > 0.05). The stance-related modulations of the SL_PO between the young and older adults were opposite for the selection of threshold value (Figure 7B).

**Table 1:** **Summary of ANOVA results for age and stance effects on synchronization likelihood of all the electrode pairs (SL_All)**.

**Threshold**	**ANOVA statistics**	***Post-hoc* analysis (Stance effect)**
0.1	Stance: *F*_(1, 22)_ = 0.10, *p* = 0.756; Age: *F*_(1, 22)_ = 0.37, *p* = 0.551; Stance × Age: *F*_(1, 22)_ = 0.20, *p* = 0.659	N.S.
0.2	Stance: *F*_(1, 22)_ = 2.64, *p* = 0.118; Age: *F*_(1, 22)_ = 0.08, *p* = 0.784; Stance × Age: *F*_(1, 22)_ = 0.32, *p* = 0.575	N.S.
0.3	Stance: *F*_(1, 22)_ = 11.04, *p* = 0.003; Age: *F*_(1, 22)_ = 0.02, *p* = 0.892; Stance × Age: *F*_(1, 22)_ = 0.93, *p* = 0.332	–
0.4	Stance: *F*_(1, 22)_ = 18.40, *p* < 0.001; Age: *F*_(1, 22)_ = 0.44, *p* = 0.515; Stance × Age: *F*_(1, 22)_ = 1.05, *p* = 0.317	–
0.5	Stance: *F*_(1, 22)_ = 18.19, *p* < 0.001; Age: *F*_(1, 22)_ = 0.32, *p* = 0.579; Stance × Age: *F*_(1, 22)_ = 0.38, *p* = 0.544	–
0.6	Stance: *F*_(1, 22)_ = 16.69, *p* < 0.001; Age: *F*_(1, 22)_ = 0.85, *p* = 0.366; Stance × Age: *F*_(1, 22)_ = 0.00, *p* = 0.974	–
0.7	Stance: *F*_(1, 22)_ = 18.31, *p* < 0.001; Age: *F*_(1, 22)_ = 1.16, *p* = 0.294; Stance × Age: *F*_(1, 22)_ = 0.31, *p* = 0.584	–
0.8	Stance: *F*_(1, 22)_ = 23.16, *p* < 0.001; Age: *F*_(1, 22)_ = 0.13, *p* = 0.727; Stance × Age: *F*_(1, 22)_ = 1.05, *p* = 0.316	–
0.9	Stance: *F*_(1, 22)_ = 23.73, *p* < 0.001; Age: *F*_(1, 22)_ = 1.14, *p* = 0.296; Stance × Age: *F*_(1, 22)_ = 0.10, *p* = 0.753	–

*N.S., non-significance*.

*−, post-hoc analysis should not be processed due to non-significant interaction effect*.

**Table 2:** **Summary of ANOVA results for age and stance effects on synchronization likelihood of the electrode pairs in the fronto-sensorimotor area (SL_FSM)**.

**Threshold**	**ANOVA statistics**	***Post-hoc* analysis (stance effect)**
0.1	Stance: *F*_(1, 22)_ = 9.24, *p* = 0.006; Age: *F*_(1, 22)_ = 4.69, *p* = 0.041; Stance × Age: *F*_(1, 22)_ = 0.96, *p* = 0.338	–
0.2	Stance: *F*_(1, 22)_ = 14.19, *p* = 0.001; Age: *F*_(1, 22)_ = 4.09, *p* = 0.056; Stance × Age: *F*_(1, 22)_ = 0.14, *p* = 0.709	–
0.3	Stance: *F*_(1, 22)_ = 16.73, *p* < 0.001; Age: *F*_(1, 22)_ = 4.34, *p* = 0.049; Stance × Age: *F*_(1, 22)_ = 0.40, *p* = 0.532	–
0.4	Stance: *F*_(1, 22)_ = 17.92, *p* < 0.001; Age: *F*_(1, 22)_ = 5.49, *p* = 0.029; Stance × Age: *F*_(1, 22)_ = 0.00, *p* = 0.974	–
0.5	Stance: *F*_(1, 22)_ = 20.18, *p* < 0.001; Age: *F*_(1, 22)_ = 5.31, *p* = 0.031; Stance × Age: *F*_(1, 22)_ = 0.13, *p* = 0.719	–
0.6	Stance: *F*_(1, 22)_ = 21.57, *p* < 0.001; Age: *F*_(1, 22)_ = 5.17, *p* = 0.033; Stance × Age: *F*_(1, 22)_ = 0.00, *p* = 0.974	–
0.7	Stance: *F*_(1, 22)_ = 24.15, *p* < 0.001; Age: *F*_(1, 22)_ = 4.92, *p* = 0.037; Stance × Age: *F*_(1, 22)_ = 0.05, *p* = 0.823	–
0.8	Stance: *F*_(1, 22)_ = 28.09, *p* < 0.001; Age: *F*_(1, 22)_ = 5.49, *p* = 0.029; Stance × Age: *F*_(1, 22)_ = 0.08, *p* = 0.778	–
0.9	Stance: *F*_(1, 22)_ = 29.23, *p* < 0.001; Age: *F*_(1, 22)_ = 5.03, *p* = 0.035; Stance × Age: *F*_(1, 22)_ = 0.06, *p* = 0.813	–

*−, post-hoc analysis should not be processed due to non-significant interaction effect*.

**Table 3:** **Summary of ANOVA results for age and stance effects on synchronization likelihood of the electrode pairs in the parietal-occipital area (SL_PO)**.

**Threshold**	**ANOVA statistics**	***Post-hoc* analysis (stance effect)**
0.1	Stance: *F*_(1, 22)_ = 3.40, *p* = 0.079; Age: *F*_(1, 22)_ = 5.70, *p* = 0.026; Stance × Age: *F*_(1, 22)_ = 2.16, *p* = 0.156	–
0.2	Stance: *F*_(1, 22)_ = 4.61, *p* = 0.043; Age: *F*_(1, 22)_ = 6.07, *p* = 0.022; Stance × Age: *F*_(1, 22)_ = 4.38, *p* = 0.048	Young: *F*_(1, 22)_ = 8.99, *p* = 0.007
		Older: *F*_(1, 22)_ = 0.00, *p* = 0.971
0.3	Stance: *F*_(1, 22)_ = 3.70, *p* = 0.068; Age: *F*_(1, 22)_ = 2.09, *p* = 0.162; Stance × Age: *F*_(1, 22)_ = 6.38, *p* = 0.019	Young: *F*_(1, 22)_ = 9.89, *p* = 0.005
		Older: *F*_(1, 22)_ = 0.18, *p* = 0.674
0.4	Stance: *F*_(1, 22)_ = 2.88, *p* = 0.104; Age: *F*_(1, 22)_ = 5.36, *p* = 0.030; Stance × Age: *F*_(1, 22)_ = 5.11, *p* = 0.034	Young: *F*_(1, 22)_ = 7.83, *p* = 0.010
		Older: *F*_(1, 22)_ = 0.16, *p* = 0.695
0.5	Stance: *F*_(1, 22)_ = 0.56, *p* = 0.463; Age: *F*_(1, 22)_ = 6.58, *p* = 0.018; Stance × Age: *F*_(1, 22)_ = 2.91, *p* = 0.102	–
0.6	Stance: *F*_(1, 22)_ = 0.17, *p* = 0.683; Age: *F*_(1, 22)_ = 6.38, *p* = 0.019; Stance × Age: *F*_(1, 22)_ = 2.28, *p* = 0.145	–
0.7	Stance: *F*_(1, 22)_ = 1.38, *p* = 0.253; Age: *F*_(1, 22)_ = 4.43, *p* = 0.047; Stance × Age: *F*_(1, 22)_ = 3.19, *p* = 0.088	–
0.8	Stance: *F*_(1, 22)_ = 2.72, *p* = 0.113; Age: *F*_(1, 22)_ = 3.09, *p* = 0.093; Stance × Age: *F*_(1, 22)_ = 4.35, *p* = 0.049	Young: *F*_(1, 22)_ = 0.10, *p* = 0.760
		Older: *F*_(1, 22)_ = 6.98, *p* = 0.015
0.9	Stance: *F*_(1, 22)_ = 3.49, *p* = 0.075; Age: *F*_(1, 22)_ = 2.42, *p* = 0.134; Stance × Age: *F*_(1, 22)_ = 4.55, *p* = 0.044	Young: *F*_(1, 22)_ = 0.04, *p* = 0.853
		Older: *F*_(1, 22)_ = 8.07, *p* = 0.009

*−, post-hoc analysis should not be processed due to non-significant interaction effect*.

**Figure 8:**
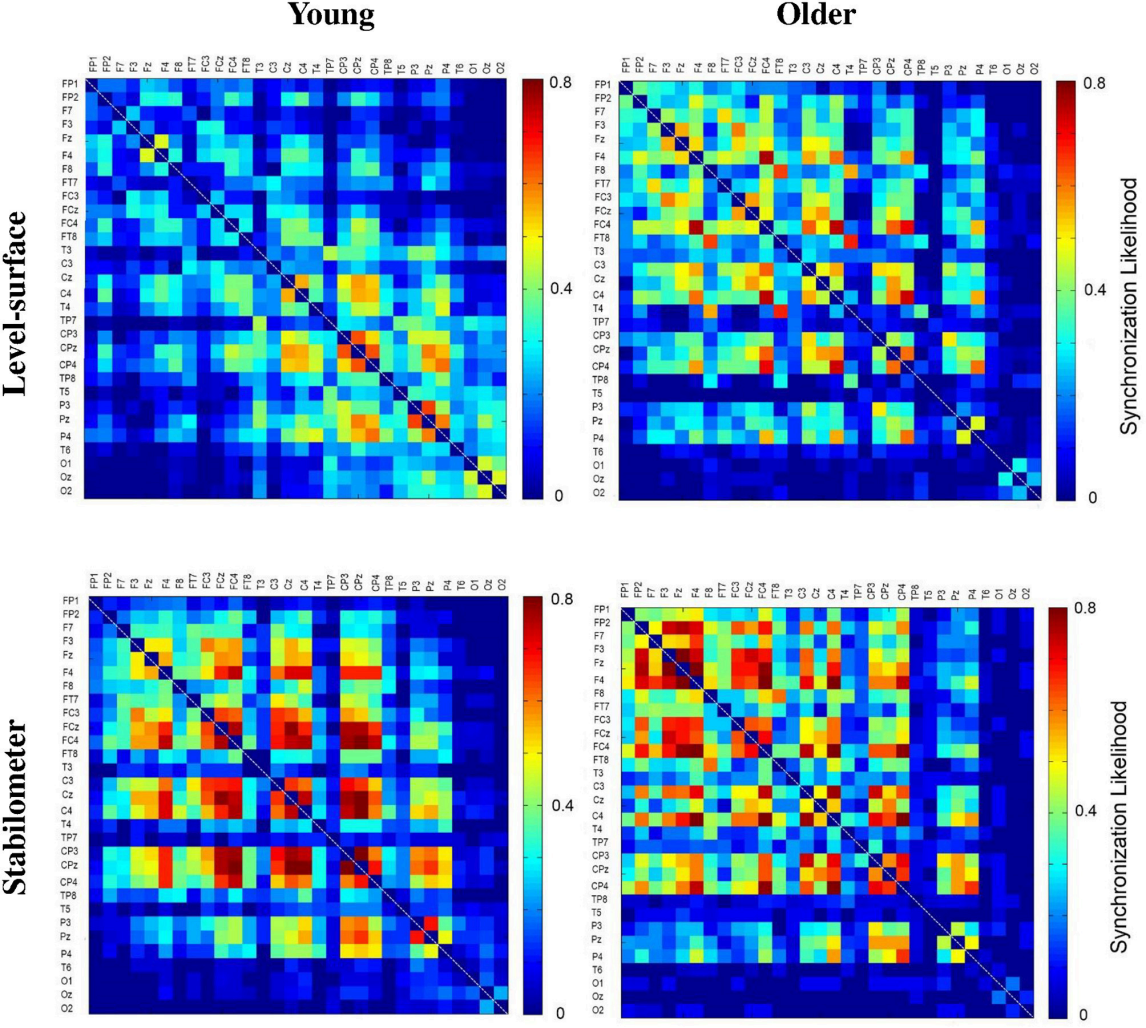
**The pooled adjacent matrix of SL of preparatory ERP (303.0 ± 8.3 ~ 326.4 ± 5.6 ms) for the concurrent force-matching and postural tasks for the young and older groups in the level-surface and stabilometer conditions (threshold value = 0.3)**.

**Figure 9:**
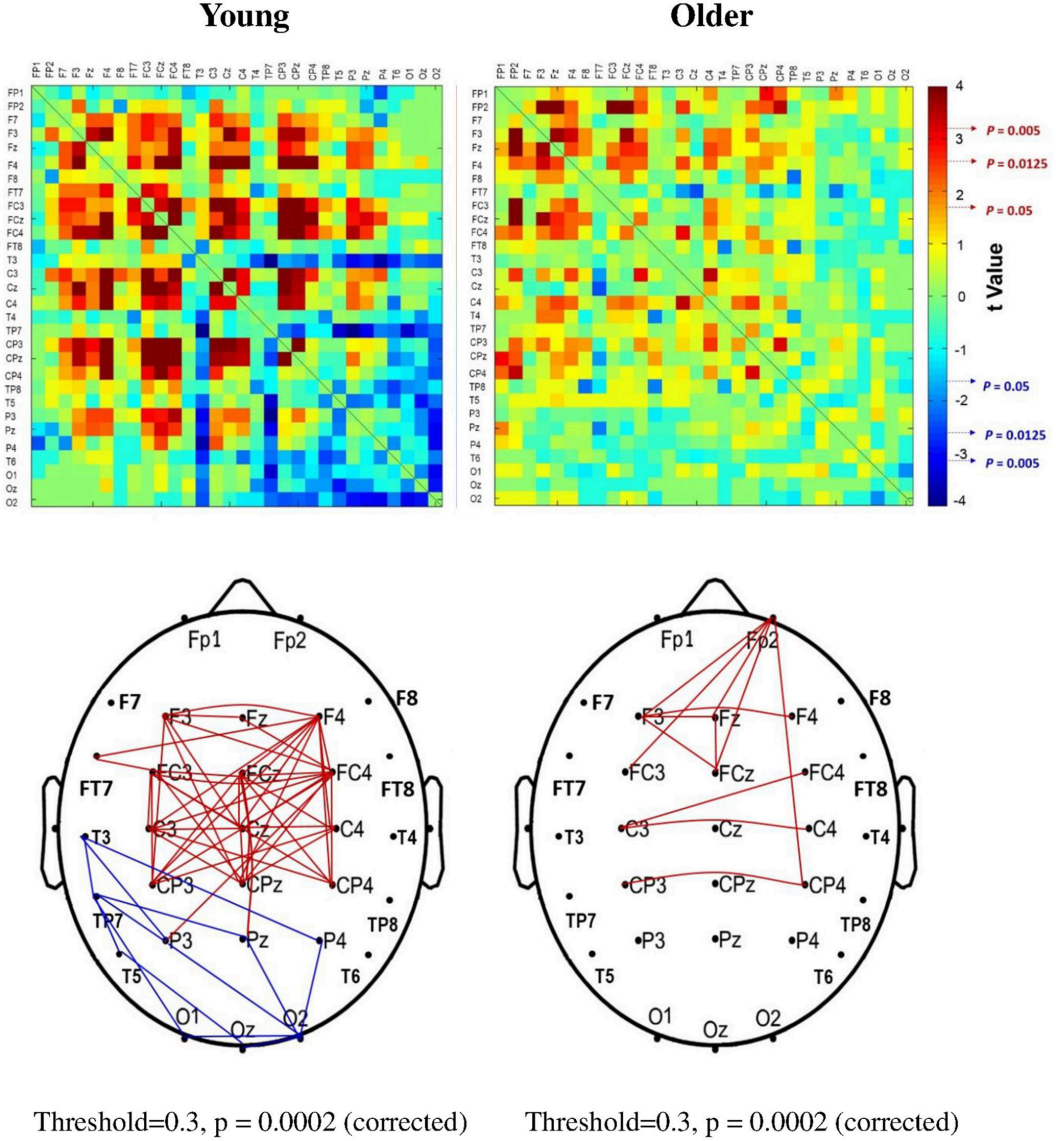
**The adjacent matrix of ***t***-values that contrasts SL between the level-surface and stabilometer conditions for the young and older groups (upper plot)**. The adjacent matrix of *t*-values clearly shows a different trend of stance-related modulation of SL across all electrode pairs for the young and older groups, (*t* > 1.771: stabilometer SL > level-surface SL, *p* < 0.05; *t* < −1.771: level-surface SL > stabilometer SL, *p* < 0.05). The lower plots display the results of connectivity analysis with network-based statistics (threshold value = 0.3). A contrasting wiring diagram shows topological distributions of the suprathreshold connectivity that vary with stance difficulty increase for the young and older groups. Red line: stabilometer connectivity of supra-threshold > level-surface connectivity of supra-threshold, *p* < 0.005; blue line: level-surface connectivity of supra-threshold > stabilometer connectivity of supra-threshold, *p* < 0.005.

The significance of spatial distribution change in SL (threshold value = 0.3) with respect to stance configuration was examined with NBS. The threshold was selected to contrast the alterations in the brain wiring diagram at relatively stronger functional connectivity. For the strongest SL with thresholds set at 0.1 and 0.2, the stance-dependent difference in SL variables was not always evident between the young and older adults (Tables 1–3). Figure 8 presents the pooled adjacent matrix of SL of preparatory ERP in the level-surface and stabilometer conditions for the young and older groups (threshold value = 0.3). The SL difference of all electrode pairs between the level-surface and stabilometer conditions was labeled with the adjacent matrix of *t*-values (*t* > 1.771: stabilometer SL > level-surface SL, *p* < 0.05; *t* < −1.771: level-surface SL > stabilometer SL, *p* < 0.05; Figure 9, upper row). The results of NBS indicated that changes in stance configuration significantly altered the brain functional connectivity in both groups (*p* = 0.0002, corrected; Figure 9, lower row). In addition, there were notable topological differences in dual task organization of supra-threshold connectivity for the young and older adults to cope with increasing postural load. The young adults in the stabilometer condition exhibited a global potentiation of supra-threshold connectivity in the fronto-sensorimotor cortex and reduction in supra-threshold connectivity between the left temporal area and the parietal-occipital cortex, as compared with the level-surface condition. In contrast, when postural load increased, the older adults enhanced supra-threshold connectivity in the fronto-sensorimotor cortex of the bilateral hemispheres and between the frontal and right prefrontal cortex. No significant suppression of supra-threshold connectivity was noted for conducting force-matching with increasing postural load in the older group.

## Discussion

The present postural-supraposatural task produced an expected outcome: the suprapostural performance and the size of postural sway of the older adults were more vulnerable to increasing postural load than those of the young adults (Figure 3). Contrary to the idea of attention withdrawal from the postural task to facilitate supraposture performance (Donker et al., 2007; Kuczyński et al., 2011), additional investment of neural resource on the postural task was necessary to prepare for concurrent force-matching with increasing postural load, in light of increased AMF_RMS and decreased AMF_SampEn for the young and older adults in the stabilometer condition. Our behavior results imply distinct brain mechanisms to cope with posture destabilization in young and older adults during a postural-suprapostural dual task. In terms of ERP connectivity analysis, the aged brain exhibited compensatory recruitment of the right prefrontal network and lack of sufficient neural economy for task switching from a postural task to a secondary force-matching act, when postural load multiplied during the stabilometer stance.

### Increase in the strength of functional connectivity for postural destabilization

The primary finding of this study was that the increase in postural load from level-surface to stabilometer stances is associated with a marked increase in global functional connectivity (SL_All) for the young and older adults (Figure 6, Table 1). Generally speaking, our data revealed the feasibility of utilizing central residual capacity for the healthy elderly to deal with stance instability during a postural-suprapostural task.

The whole fronto-parietal network is integrated to coordinate a postural-suprapostural task (Karim et al., 2013; Ferraye et al., 2014). On account of a stance-related increase in functional connectivity of the fronto-sensorimotor network (SL_FSM) for both the young and older adults (Figure 7A, Table 2), it was plausible that force-matching from stabilometer stance caused a shift to a state in which the frontal control predominated, linking to increase in attentional demand to posture stabilization. In fact, several previous studies reported a parallel enhancement of cortical recording from the frontal cortex and supplementary motor area following posture perturbation (Mihara et al., 2008; Fujita et al., 2016). Also, the stabilometer fluctuation movements aggravated externally-induced retinal image motion, so as to hampered precise visual target location (Sipp et al., 2013; Hülsdünker et al., 2015) and then enhance mid-frontal activity for action monitoring and error processing prior to force-matching (Mihara et al., 2008). The stance-dependent increases in SL_FSM also accounted for the unexpected lack of an increase in N1 amplitude in the stabilometer condition. Our previous work on a posture-motor task revealed that force-matching from unipedal stance led to a greater N1 amplitude than force-matching from bipedal stance (Huang and Hwang, 2013). Originated in the fronto-central region (Adkin et al., 2008), N1 amplitude reflects monitoring of the attentional states (Huang and Hwang, 2013; Huang et al., 2014) and sensory processing (Sibley et al., 2010) of postural perturbation in a postural-suprapostural task. The insignificant variation in N1 amplitude in this study may partly due to the use of a stabilometer of low curvature that did not produce as much stance instability as unipedal stance would have. Moreover, the most appealing explanation to reconcile the paradoxical finding is that a dual task may not necessarily alter regional activation, instead altering the interactions of the frontal/prefrontal areas with other cortical regions [such as parietal (Gontier et al., 2007) and premotor areas (Marois et al., 2006)]. Of note, the older adults exhibited a stronger SL_FSM than the young adults (Figure 7A, Table 2). Although, the older adults seemingly recruited more central resource in the fronto-sensorimotor network in the both stance conditions, yet it could not nicely explain why dual-task performance of the older adults tended to be more vulnerable to higher postural load.

### Lack of neural economy in the elderly

The interaction effect of age and stance configuration of SL_PO plays a critical role in age-dependent differences in dual-task performance with increasing postural load. The young adults showed surprising desynchronization of the PO network, in view of the decline in SL_PO with increasing postural load at the threshold values of 0.2, 0.3, and 0.4 (Figure 7B, Table 3). During concurrent execution of force-matching in the stabilometer condition, the young adults appeared to avoid division of attentional resources toward multisensory information by dissociating the neuro-anatomical implementation in the PO network. As stabilometer stance did not cause inferior force-matching performance in the young adults (Figure 3), the scenario suggests neural economy (Schubert, 2008) or adaptive resource sharing (Mitra and Fraizer, 2004), with which the young adults could minimize the dual-task cost to facilitate task switching for the subsequent force-matching event (Huang et al., 2016). In contrast, the older adults increased the weak functional connectivity (threshold value = 0.9) of the PO network in the stabilometer condition (Figure 7B, Table 3). The genesis of the relatively weak connectivity simply taxed a limited central resource from the aged brain, because there was no significant performance benefits associated with increasing postural load for the older adults (Figure 3).

Further supporting the notion of age-related deficits in neural economy for a postural-suprapostural task is the topology of the wiring diagram (Figure 9). The left temporal lobe is known to handle the timing of complex movements with auditory cues (Nakai et al., 2005). Previous fMRI studies revealed that the superior temporal sulcus and posterior middle temporal gyrus of the left hemisphere are more selective to body actions and actions performed on other objects, respectively (Jellema and Perrett, 2006; Assmus et al., 2007). Studies of the macaque monkey (Perrett et al., 1989; Jellema and Perrett, 2003) have shown the superior temporal sulcus to be modulated by body posture during target reaching. Hence, the functional connectivity of the left temporal-parietal-occipital network (TPO network) in a postural-suprapostural task might serve to identify the execution beep (distinguishing the tone from the warning signal) and integration of sensory information from the parietal cortex regarding body schema representation (Pellijeff et al., 2006) as well as stance fluctuations (Noppeney et al., 2005; Vangeneugden et al., 2011). For the young adults, multimodal sensory integration to detect postural instability using the TPO network was conditionally disengaged before force-matching. In that brief moment, postural control could be temporarily regulated by automatic responses using postural synergy in the midbrain (Jacobs and Horak, 2007). The suppression of information transfer in the left TPO network would augment resource availability upon retrieval of spatial information of the visual target for force-matching (Chelazzi et al., 1993; Kastner and Ungerleider, 2001), such as occurs in solving task conflicts (Schall et al., 2002; Schulz et al., 2011) and facilitating task-switching from stabilometer stance to force-matching (Hwang and Huang, 2016) using frontal executive function. In fact, changes in P2 amplitude with respect to stance configuration also support the argument that the elderly paid less attention to force-matching preparation. In experiments of force-matching to couple a static or a dynamic target, P2 amplitude is inversely related to attentional focus on the visual target of the force-matching task (Huang and Hwang, 2013; Huang et al., 2014). Hence, greater P2 amplitude in the stabilometer condition (Figure 5, the right column) might indicate that the participants did not focus well on the force-matching event during posture challenge, especially in older adults.

A slower adaptive process for the aged brain is an alternative explanation for why control hubs in the TPO network of the older adults was not dissociated with high postural load. According to the free energy principle (Friston et al., 2006; Friston, 2010), the predictive coding is generated by communication with actual sensory feedback connections to update cortical representations on a trial-by-trial basis (Panichello et al., 2013). When the incoming sensory information coincides with the predictive coding, free energy is minimized. Instead, the brain keeps estimating most-likely likelihood from the information changes in the sensory feedback with environment contexts. For those young adults who could more quickly adapt to stabilometer stance before force-matching, the left TPO network was less activated for a small prediction error when descending prediction efficiency interpreted the actual sensory input. A natural consequence of aging causes slow adaptation and deviance detection of environmental changes. For our older cohort, the TPO network in the stabilometer condition was not suppressed, because they still kept reinforcing internal generative model by comparing of predictions and actual sensory inputs (primarily the ventral visual sources) till free energy was optimally minimized (Panichello et al., 2013).

### Compensatory recruitment of the right prefrontal network in the elderly

Unlike the young adults, the older adults revealed stance-related enhancements of functional connectivity between the right pre-frontal and frontal areas (Figure 9, right). In contrast to the level-surface condition, the associated force-matching with the stabilometer stance recruited more attentional resources to deal with increases in postural sway and stance-induced difficulty in target detection prior to force-matching. Recently, the prefrontal area has been linked to balance control, especially when unexpected external postural perturbation is provided (Maki and McIlroy, 2007; Mihara et al., 2008). In healthy adults and stroke patients, the right prefrontal lobe plays a greater role than the left prefrontal lobe in stance control during postural perturbation (Ugur et al., 2000; Fujita et al., 2016). Prefrontal lateralization is related to resetting eye positions in accordance with using spatial working memory processes (Mihara et al., 2008; Fujita et al., 2016). The dorsolateral prefrontal cortex was also found to play a critical role in goal-directed behavior (de Wit et al., 2009), integrating environmental contexts with information on body positioning of the elderly (Wang et al., 2016). The prefrontal area, which receives cerebellum influences via the thalamus (Middleton and Strick, 2001), has dense projections to the pontine nuclei (Ramnani et al., 2006) for reflexive control of postural balance following external stance perturbation (Hartmann-von Monakow et al., 1981; Mihara et al., 2008). Previous behavioral studies have shown that the elderly often prioritize attentional allocation to posture maintenance above supraposture performance with increasing stance destabilization, known as a “posture-first strategy” (Shumway-Cook and Woollacott, 2000; Doumas et al., 2008). Hence, the age-dependent compensatory recruitment of the right prefrontal lobe is in a good agreement with the greater need for spatial attention of the elderly adults in the postural-suprapostural task with increasing postural load. The prevailing attentional focus on the posture subtask of the elderly added to the difficulty in task-switching at the cost of inferior force-matching performance. In fact, for healthy cognitive aging, additional recruitment of cognitive processes mediated by the prefrontal cortex and its vast interconnections were found to increase with task demand (Allali et al., 2014; Toepper et al., 2014), in accordance with the compensation-related utilization of neural circuits hypothesis (Reuter-Lorenz and Cappell, 2008).

### Methodology issues

To date, SL has most commonly been used to characterize multiple synchronized neural sources in the brain (Stam and van Dijk, 2002) using low-density (Smit et al., 2012; Herrera-Díaz et al., 2016) or high-density EEG (Polanía et al., 2011). The major methodological advantage of using SL is that it can sensibly detect slight and intricate variations in the coupling strength (Koenis et al., 2013), resolving rapid synchronization patterns in the non-stationary ERP profile at a short time scale (Stam and van Dijk, 2002; Betzel et al., 2012). Some simulation studies have argued that SL could bring about spurious coupling due to a volume conduction effect (Stam et al., 2005; Tognoli and Kelso, 2009). The authors agree that an increase in postural challenge probably led to enhanced volume conduction for the recruitment of more neurons in the stabilometer condition, especially for those functional connectivity grouped with neighboring regions. If the physical synchronization did exist, we could not completely deny overestimation of the functional connectivity within neighboring electrodes. However, physical synchronization does not rationally explain our major finding of age-related differences in connectivity reorganization with increasing postural load. In contrast to the young adults, the older adults did not exhibited a polarization modulation of spatially-distributed communities for the FSM and TPO networks with increasing postural load (Figure 9). Particularly for the stronger functional connectivity, the stance-related modulations of the two distinct networks in young adults and lack of the paralleling connectivity change in the elderly were hard to reconcile with the global rise or fall of state transition to intermittent activity of a single common-source volume conduction. Moreover, we did not intend to specify any age-related differences in functional connectivity of neighboring electrodes or small-range excitability of a recording electrode, as we did accentuate age-dependent parametric changes on a network basis (Figures 5–7). Although, some measures of functional connectivity, such as phase lag index, have been proposed to minimize common sources (Stam et al., 2007), phase-based approaches that can be more sensitive to noise measure temporal-spatial properties of functional connectivity that are quite different from SL (Vinck et al., 2011). Phase-based approaches are not appropriate for accessing the inter-dependences of two short-length ERP profiles in the presence of non-stationarities (Cohen, 2015). As no quantification of functional connectivity is perfect, future work may consider a combination of local cerebral hemodynamic properties and spontaneous neural activity. However, it is not feasible to fully rule out potential common sources with the present setup.

## Conclusion

At the neural level, the present work first reveals neural underpinnings which are associated with (but not necessarily causal to) why dual-task performance of the older adults is tended to be more easily affected by postural load increment. In comparison with the young adults, high postural load produced more dual-task cost of the older adults, pertaining to lack of neural economy to timely deactivate the left TPO network. In addition, the age-dependent compensatory recruitment of the right prefrontal lobe indicates that the older adults may need a greater spatial attention for dual-task control against fluctuating stabilometer movements.

## Ethics statement

National Cheng Kung University Hospital Research Ethics Committee. A physical therapist explained the study purpose and experiment procedure for each participant. All participants gave informed consent to participate according to a protocol approved by the local ethics committee (University Hospital, National Cheng Kung University, Taiwan). There is no vulnerable populations in the present study.

## Author contributions

Substantial contributions to the conception or design of the work or the acquisition: CH, LL, and IH. Analysis or interpretation of data for the work: CH and IH. Drafting the work or revising it critically for important intellectual content: CH and IH. Final approval of the version to be published and agreement to be accountable for all aspects of the work in ensuring that questions related to the accuracy or integrity of any part of the work are appropriately investigated and resolved: CH, LL, and IH.

## Funding

This research was supported by a grant from the Ministry of Science and Technology, Taiwan, ROC, under grant no. MOST 103-2314-B-002-007-MY3 and MOST 104-2314-B-006-016-MY3.

### Conflict of interest statement

The authors declare that the research was conducted in the absence of any commercial or financial relationships that could be construed as a potential conflict of interest.

## Abbreviations

NFE: normalized force error
RT: reaction time
SampEn: sample entropy
AMF_RMS: root mean square value of ankle fluctuation movements
AMF_SampEn: sample entropy of ankle fluctuation movements
GF: global frontal
SM: sensorimotor
PO: parietal-occipital
SL: synchronization likelihood
NBS: network-based statistics
FSM: fronto-sensorimotor
TPO: temporal-parietal-occipital.
